# Revealing a Mutant-Induced Receptor Allosteric Mechanism for the Thyroid Hormone Resistance

**DOI:** 10.1016/j.isci.2019.10.002

**Published:** 2019-10-02

**Authors:** Benqiang Yao, Yijuan Wei, Shuchi Zhang, Siyu Tian, Shuangshuang Xu, Rui Wang, Weili Zheng, Yong Li

**Affiliations:** 1 State Key Laboratory of Cellular Stress Biology, Innovation Center for Cell Signaling Network, School of Life Sciences, Xiamen University, Fujian 361005, China

**Keywords:** Biological Sciences, Molecular Biology, Structural Biology

## Abstract

Resistance to thyroid hormone (RTH) is a clinical disorder without specific and effective therapeutic strategy, partly due to the lack of structural mechanisms for the defective ligand binding by mutated thyroid hormone receptors (THRs). We herein uncovered the prescription drug roxadustat as a novel THRβ-selective ligand with therapeutic potentials in treating RTH, thereby providing a small molecule tool enabling the first probe into the structural mechanisms of RTH. Despite a wide distribution of the receptor mutation sites, different THRβ mutants induce allosteric conformational modulation on the same His435 residue, which disrupts a critical hydrogen bond required for the binding of thyroid hormones. Interestingly, roxadustat retains hydrophobic interactions with THRβ via its unique phenyl extension, enabling the rescue of the activity of the THRβ mutants. Our study thus reveals a critical receptor allosterism mechanism for RTH by mutant THRβ, providing a new and viable therapeutic strategy for the treatment of RTH.

## Highlights

• We identified a novel THR ligand that effectively binds to THRβ mutants
• Structures revealed mechanisms for the RTH controlled by a key residue switch
• Roxadustat retains unique hydrophobic interactions with THRβ mutants
• We provide a promising approach to design THR ligands in treating RTH

## Graphical Abstract

**Figure:**
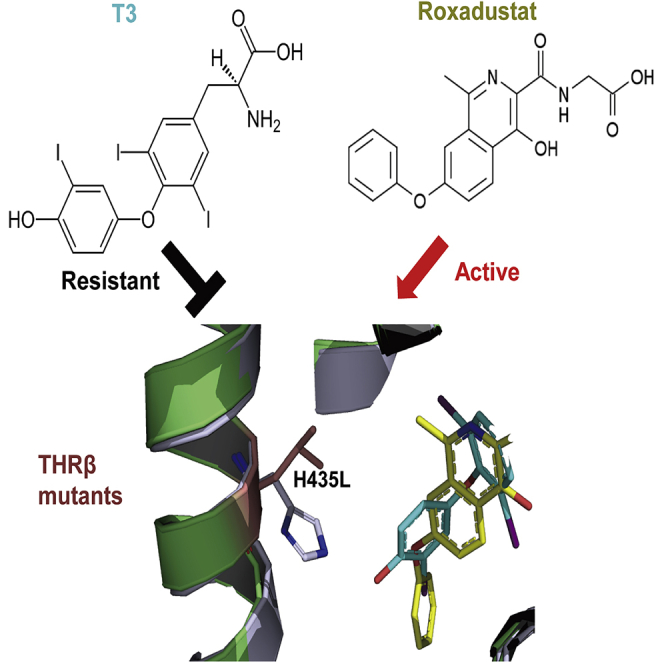

## Introduction

The thyroid hormone receptors (THRs) are nuclear hormone receptors regulated by endogenous thyroid hormones, including the inactive prohormone thyroxine (T4) and the bioactive hormone 3,3′,5-triiodothyronine (T3), which are critical for development regulation and metabolic homeostasis. Encoded by two different genes, THRα and THRβ are two main receptor subtypes with overlapping and differential characteristics in tissue distribution, ligand binding, and biological functions (Cheng et al., 2010). Notably, small molecules with THRβ subtype-selective binding activity are of great value for clinical purposes for their beneficial effects on cholesterol (Baxter and Webb, 2009). Resistance to thyroid hormone (RTH) is a clinical disorder with impaired sensitivity to thyroid hormones at the cellular and tissue level, characterized by elevated thyroid hormone level and a normal or slightly increased thyroid-stimulating hormone level, leading to variable degrees of mental and growth abnormalities (Ortiga-Carvalho et al., 2014, Refetoff et al., 1993). Although the defects in any of the processes in thyroid hormone transport and synthesis can all contribute to RTH, in most cases the disorder involves defective thyroid hormone receptors, resulting in reduced T3 binding and disruptive thyroid hormone signaling (Dumitrescu and Refetoff, 2013). Most RTH mutations identified are located in the ligand-binding domain (LBD) of THRβ, leading to resistance to thyroid hormone β (RTHβ). Although the elevated thyroid hormone levels associated with the mutations in THRβ or the applications of thyroid hormone analogues can compensate the defective THRβ activity, the excess ligands may lead to the over-stimulation of THRα associated with more severe impairment, emphasizing the importance of the development of THRβ-selective ligands in treating RTH (Wagner et al., 2001, Martinez et al., 2009). Despite much encouraging progress in developing thyroid hormone analogues for the treatment of RTHβ (Hassan and Koh, 2008), their further development and clinical application have been limited by variations in treatment outcomes, selective mutation distributions, or tissue toxicity (Groeneweg et al., 2017). Furthermore, the wide distributions of the THRβ mutation sites suggest diverse mechanisms for RTHβ and their specific therapeutic strategy accordingly (Huber et al., 2003). As such, the development of novel ligands with preferential affinity to THRβ while targeting individual THRβ mutants is of the utmost importance for the treatment of RTHβ.

The negative feedback loop of the hormone active form T3 through binding to THRs plays important roles in the thyroid hormone homeostasis (Chiamolera and Wondisford, 2009). As ligand-regulated nuclear receptors (Mangelsdorf, 1995), THRs have a structurally conserved LBD that allows the binding of distinct ligands (Burris et al., 2013). The binding of ligands is regulated by a combination of hydrophobic and hydrophilic interactions with the residues in the ligand-binding pocket located in the receptor LBD. Following the ligand binding, the function of THRs is mediated through the selective recruitment or release of specific coregulators, like the family of steroid receptor coactivators (SRCs) (Li et al., 2003, Jin and Li, 2010, Savkur and Burris, 2004), as a heterodimeric complex with retinoid X receptor (RXR) (Putcha et al., 2012, Kojetin et al., 2015, Mangelsdorf and Evans, 1995). Moreover, since ligand binding and ligand-mediated cofactors recruitment are crucial for functions mediated by THRs, the LBD and the ligand-binding pocket have been the focus of intensive structural study, providing the molecular basis for the thyroid hormone binding and receptor subtypes selectivity. However, the precise molecular mechanisms underlying the defective thyroid hormone binding by various mutant receptors remain unclear. The structural insights into the defective ligand binding by THRβ mutants will be imperative for the rational design of effective mutant-specific ligands for the treatment of RTH.

## Results

### Identification of Roxadustat as a THRβ-Selective Ligand

**Figure 1:**
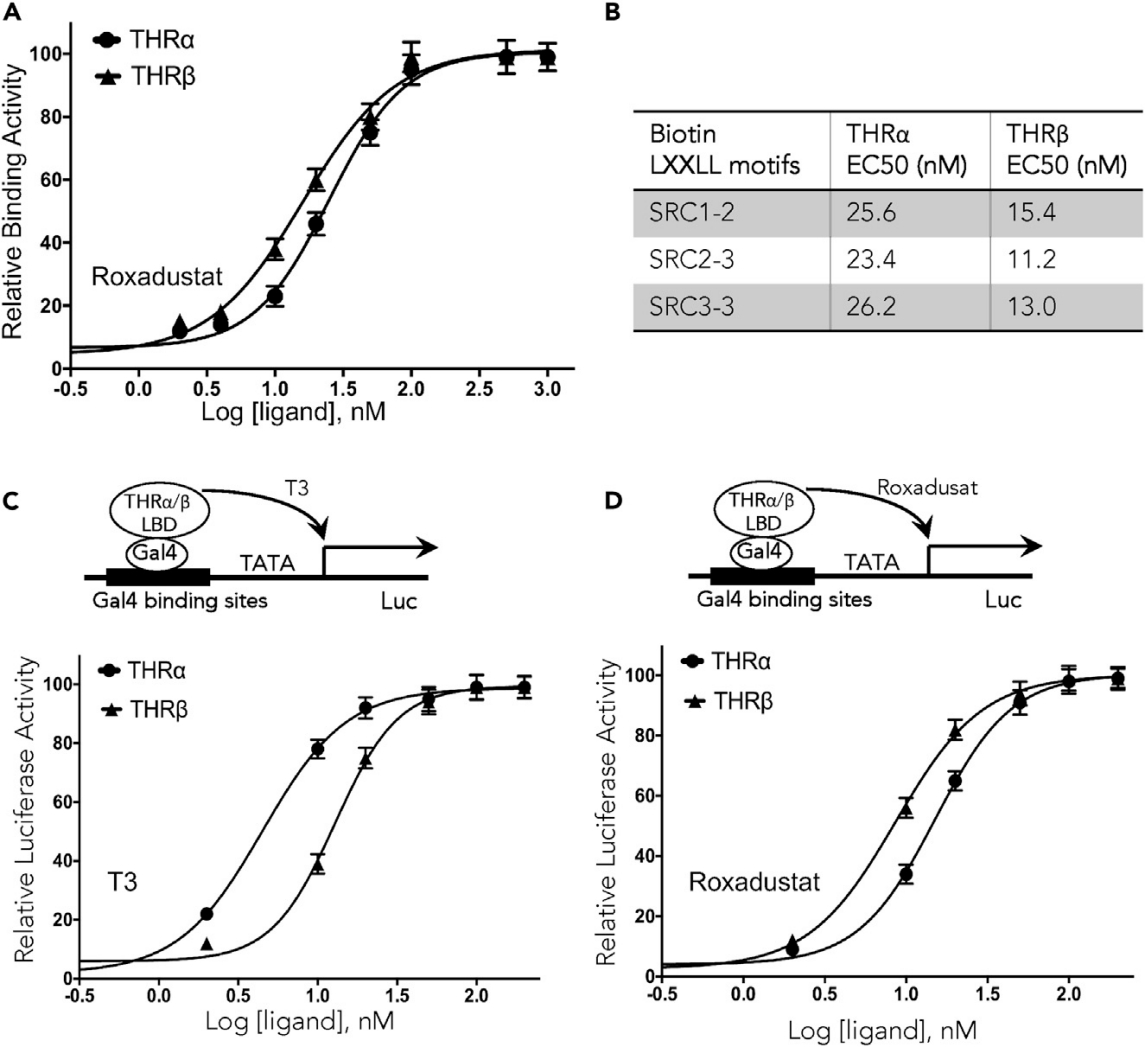
THR Subtype-Selective Binding and Activity of Roxadustat (A) Dose-response curves of the THRα and THRβ LBDs to recruit the coactivator SRC1 motif in response to roxadustat measured by AlphaScreen assays. (B) The EC50 values of various co-activator peptide motifs to the THRα and THRβ LBDs in response to roxadustat by AlphaScreen assays. (C and D) Dose-response curves of the transactivation activity of the THRα and THRβ LBDs in response to T3 (C) or roxadustat (D) by cell-based luciferase assays, respectively. Values are the means ± SD of three independent values.

In search of novel ligands for THRs, we used THRβ LBD as bait to screen chemical libraries based on AlphaScreen biochemical assay, which determines the efficacy of small molecules in recruiting coregulator peptides to the THRβ LBD. Surprisingly, roxadustat (FG-4592), a first-in-class oral hypoxia-inducible factor prolyl hydroxylase (HIF-PH) inhibitor (Bouchie, 2013) and recently approved drug for the treatment of anemia, was revealed as a positive THRs activator. With a molecular scaffold distinct from native thyroid hormones (Figure S1), roxadustat strongly promoted the interaction of both THRα and THRβ with various coactivator LXXLL motifs from the family of steroid receptor coactivators (SRC1, SRC2, and SRC3) with EC50s of about 25 and 15 nM, respectively (see Transparent Methods and Figures S2, 1A, and 1B), suggesting an agonist nature of the ligand. In agreement with AlphaScreen results, cell-based mammalian one-hybrid reporter assay was performed to confirm the efficacy of roxadustat in activating THRs in mammalian cells (Figures 1C and 1D), further affirming that roxadustat is a highly potent THRs ligand with biological functions. Interestingly, both AlphaScreen and reporter assay indicated that roxadustat interacts with THRβ with higher potency compared with THRα (Figure 1), suggesting a THRβ-selective binding nature of roxadustat.

### Structural Basis for the Recognition of Roxadustat by THRβ LBD

**Figure 2:**
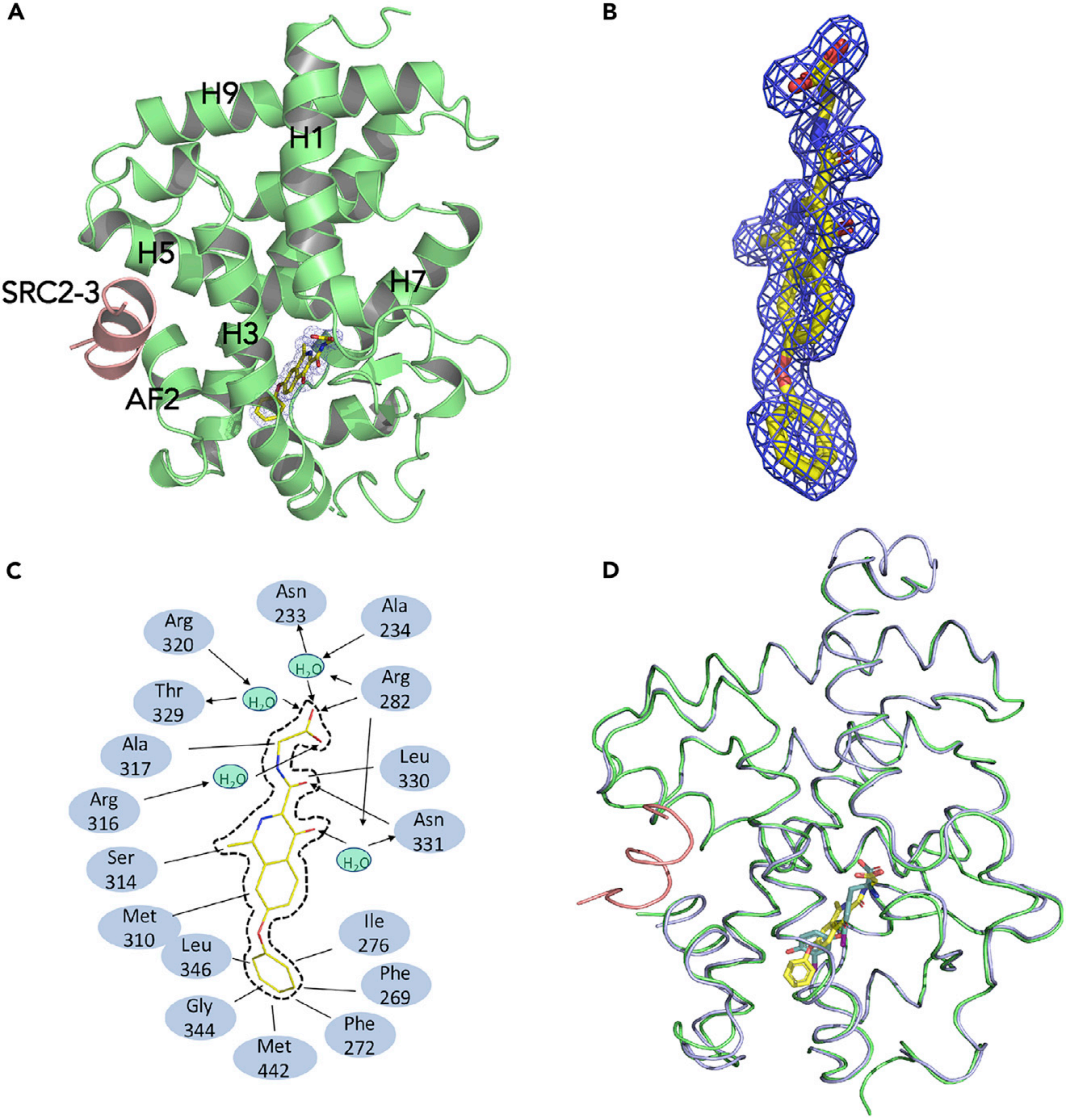
Structural Analysis of the Recognition of Roxadustat by THRβ LBD (A) The structure of roxadustat bound with THRβ LBD in cartoon representation. THRβ LBD is colored in lime, and the SRC2-3 motif is in salmon. The bound roxadustat is shown in stick representation with carbon, nitrogen, and oxygen atoms depicted in yellow, blue, and red, respectively. (B) A 2Fo-Fc electron density map (1.0 δ) showing the bound roxadustat. (C) Schematic representation of the roxadustat-THRβ interaction. The arrows and the lines represent the hydrogen bonds and hydrophobic interactions between ligand and LBD residues, respectively. (D) Superposition of the T3-bound THRβ (light blue) with the roxadustat-bound THRβ (lime), where T3 is in cyan and roxadustat is in yellow.

To determine the molecular basis of the binding selectivity of roxadustat to THRβ, we solved the crystal structures of THRβ LBD complexed with roxadustat (Table S1). The structure reveals that the roxadustat-bound THRβ adopts a canonical active conformation in a three-layer helical sandwich arrangement that resembles most agonist-bound nuclear receptor structures (Figure 2A), in agreement with the agonist activity of roxadustat on THRs by both AlphaScreen and reporter assays (Figures S2 and 1). The existence of roxadustat was apparent from the highly revealing electron density map shown in Figure 2B, whose interaction with THRβ was stabilized by a combination of hydrogen bonds and hydrophobic interactions (Figure 2C). Superposition of the roxadustat-bound THRβ structure with the T3-bound THRβ (PDB ID 3GWS) (Nascimento et al., 2006) showed that roxadustat aligned well with the native ligand T3 and occupied the similar binding site in the THRβ pocket (Figure 2D). Notably, structural alignment of THRβ-bound roxadustat with various THR ligands available at PDB revealed that roxadustat shares a conserved carboxyl head group for all the THR ligands (Figure S3) (Nascimento et al., 2006, Sandler et al., 2004, Huber et al., 2003, Dow et al., 2003, Ye et al., 2003, Borngraeber et al., 2003, Hangeland et al., 2004, Bleicher et al., 2008, Koehler et al., 2006). Surprisingly, instead of a conserved hydrophilic hydroxyl group at the tail shared by all the other THR ligands, roxadustat has a hydrophobic phenyl extension at the corresponding site, which results in mainly hydrophobic interactions with THRβ at this position (Figure 2C), suggesting a unique functional nature of roxadustat as a THR ligand.

### Structural Basis for the THRβ Selectivity by Roxadustat

**Figure 3:**
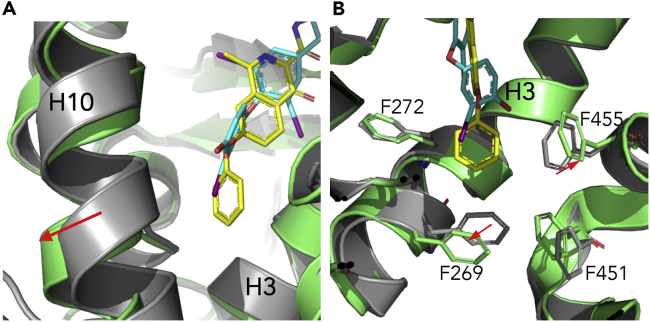
Structural Basis for the THRβ Selectivity by Roxadustat A structural alignment of the structures of T3/THRα LBD (gray) and roxadustat/THRβ LBD (lime) is shown in cartoon representations with T3 and roxadustat shown in cyan and yellow, respectively. Differential conformations of the backbone (A) and individual residue side chains (B) between THR α and THRβ LBD that contribute to the receptor selectivity are indicated by red arrows.

A structural alignment of the roxadustat-bound THRβ structure with the T3-bound THRα (PDB ID 2H77) (Nascimento et al., 2006) revealed a structural mechanism for the THRβ receptor selectivity by roxadustat (Figure 3). Despite that roxadustat overall aligns well with T3, a unique structural feature of roxadustat different from T3 is its extended hydrophobic phenyl group, suggesting that a larger hydrophobic cavity is needed for the effective ligand binding. Interestingly, the helix 10 of THRβ shifts outward to make extra space for the phenyl extension of roxadustat in comparison with that of THRα (Figure 3A). Similarly, the side chains of the hydrophobic residues, such as F272 and F269 of THRβ, display differential conformations compared with their corresponding residues of THRα, resulting in a larger pocket size to accommodate the binding of roxadustat (Figure 3B). In summary, differential conformations between THRβ and THRα on the backbone and individual residue side chains coupled with the unique larger phenyl extension of roxadustat contribute to the THR subtype selectivity of roxadustat, highlighting the differential roles of THR pocket residues in recognizing various ligands.

### Roxadustat Overcomes Thyroid Hormone Resistance Caused by Receptor Mutations

**Figure 4:**
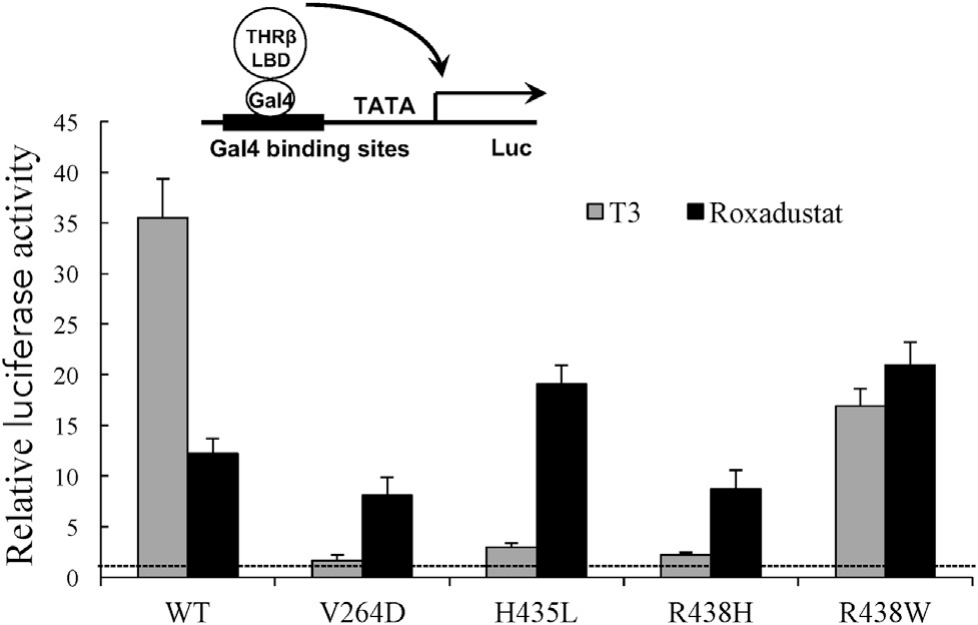
Roxadustat Overcomes Thyroid Hormone Resistance Caused by Thyroid Hormone Receptor Mutations The fold induction of transcriptional luciferase activity by THR ligands in reporter assays. 293T cells were co-transfected with pG5Luc reporter together with the plasmids encoding various THRβ mutant LBDs fused with the Gal4 DNA-binding domain. After transfection, cells were treated with DMSO or 2 μM T3 or 2 μM roxadustat. The dashed line indicates one-fold activation by the ligands compared with the DMSO treatment. Values are the means ± SD of three independent values.

Given the unique nature and scaffold of roxadustat as a THRβ-selective ligand, we next investigated its ability in activating THRβ associated with thyroid hormone resistance. As shown in Figure 4, treatment with roxadustat significantly induced the transcriptional activity of wild-type THRβ in Gal-4 driven reporter assays, to a lesser extent than that of T3. As expected, the native ligand T3 abolished or substantially diminished the transcriptional activity of four THRβ mutants associated with thyroid hormone resistance, V264D, H435L, R438H, and R438W, respectively (Wakasaki et al., 2016, Nomura et al., 1996, Sabet and Pallotta, 2011, Narumi et al., 2010). Surprisingly, the treatment of roxadustat either enhanced or retained the transcriptional activity of these THRβ mutants, all leading to higher induced transcriptional activity than those of T3 (Figure 4). The thermal stability analysis showed that both T3 and roxadustat increased the thermal stability of TRβ wild-type, with a higher Tm value for the T3 treatment (Figure S4). In contrast, the binding of roxadustat resulted in higher Tm values for four TRβ mutants than that of T3 (Figure S4). All these results suggest a potential advantage of roxadustat over native thyroid hormones in treating thyroid hormone resistance caused by specific mutations.

### Structural Mechanism for the Roxadustat in Rescuing Thyroid Hormone Resistance

**Figure 5:**
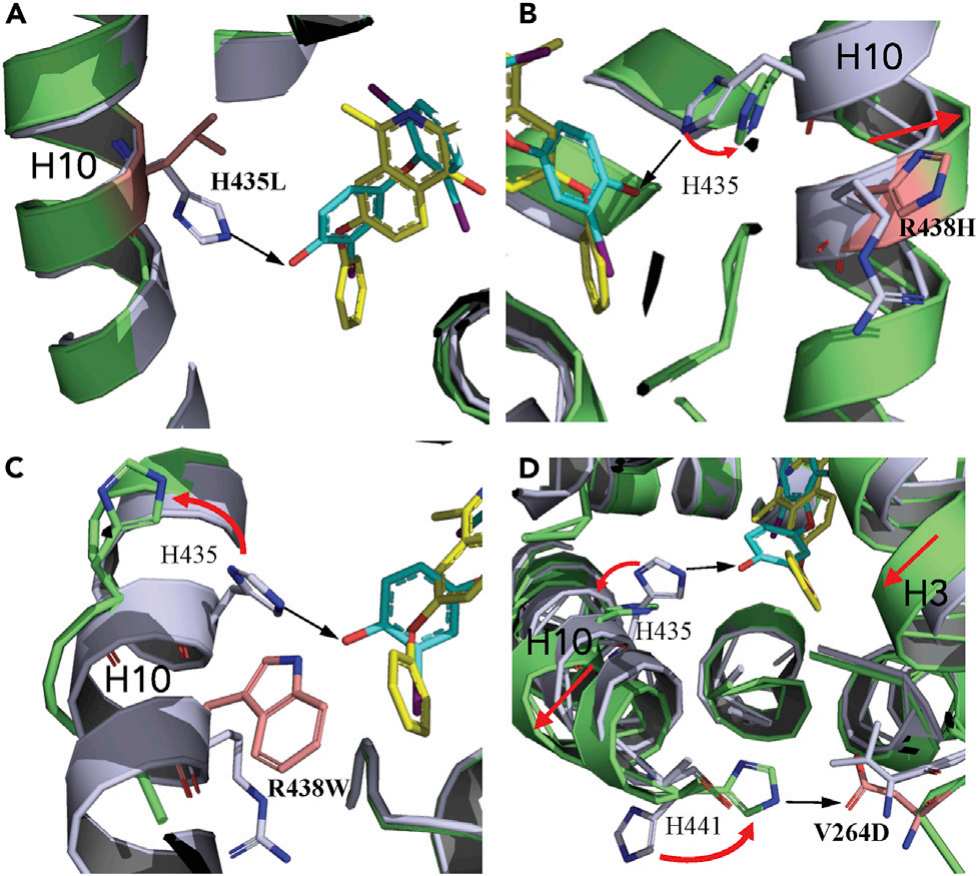
Structural Mechanism for the Roxadustat to Activate the THRβ Mutants Associated with Thyroid Hormone Resistance (A–D) Overlays of T3-bound THRβ WT (light blue) with the roxadustat-bound THRβ mutants (lime) shown as cartoon representations, with T3 and roxadustat shown in cyan and yellow, respectively. The conformational changes of the residues involved in the thyroid hormone resistance by THRβ mutations are indicated by red arrows. The mutant residues are shown in salmon red.

Although all four mutant residues are located in the THRβ LBD, H435 is the only one that directly contacts ligands T3 or roxadustat, whereas the rest of the mutant residues are located outside the ligand-binding pocket, not in the range to form intimate contacts with the ligands (Figure S5), which makes us wonder how these mutants influence the ligand binding. To unravel the molecular basis for the RTH of the THRβ mutants and further their beneficial susceptibility to roxadustat, we performed structural studies on the THRβ mutants complexed with roxadustat. The data statistics and the refined structures are summarized in Table S1. For all four THRβ mutants whose crystal structures have been solved as shown in Figure S6, roxadustat was clearly observed to fit in the electron density maps in the ligand-binding pockets. The structural analysis further revealed an apparent conformational change of residue H435 on helix 10 of THRβ for all four mutants, resulting in the loss of a critical hydrogen bond between THRβ and a critical hydroxy group of T3 (Figure 5), which is also conserved for the other THR ligands identified so far (Figure S3), explaining the severely reduced THRβ activity by T3 (Figure 4). Specifically, in addition to the direct mutation of H435 to the hydrophobic leucine of THRβ H435L (Figure 5A), the allosteric conformational changes of THRβ H435 were coupled with several intermediate structural changes, predominantly in helix 10, which were initiated by mutants R438H, R438W, or V264D, consequently leading to impaired T3 binding (Figures 5B–5D). Notably, both R438H and R438W are located on the helix 10, thereby directly affecting its conformation (Figures 5B and 5C). Although the mutant V264D is located far away at the end of helix 3, the residue Asp that resulted from the mutation provides a hydrogen bond donor to create a new hydrogen bond with H441 on the helix 10, which serves as transducer to relay conformation changing information to the helix 10 and H435 (Figure 5D).

In contrast to the reduced binding of T3 by THRβ mutants due to the loss of a critical hydrogen bond donor/acceptor contributed by H435, roxadustat retains the effective binding activity (Figure 4) owing to the hydrophobic interactions with THRβ mutants via its unique phenyl extension at the corresponding position, which was unaffected by the conformational changes of the polar H435 residue (Figure 5). The structures therefore have uncovered the mechanisms for the roxadustat in rescuing the activity of the THRβ mutants, highlighting the critical roles of the unique nature of the hydrophobic interaction between roxadustat and THRβ mutants (Figure 5). Collectively, the THRβ mutants we studied dictate the discrimination between T3 and roxadustat via the modulation of the conformation of a critical residue H435 either directly or allosterically, resulting in differential effects of the mutant receptors to native thyroid hormones and roxadustat.

## Discussion

Despite the physiological and pharmaceutical importance of RTHβ, there is still specific therapeutic strategy available, partly owing to the limited understanding of the structural mechanisms for the defective ligand binding by THRβ mutants. Indeed, the lack of ligands that efficiently bind THR mutants makes the structural study of RTH difficult, given the critical roles of the ligand binding in the stabilization of THRs for biochemical and structural studies, which is evidenced by the unavailability of any apo-THR crystal structures. Here, we report the identification of an anemia drug roxadustat as a novel modulator for THRs with a therapeutic potential in the treatment of RTH, thereby providing a small molecule tool enabling the first probe into the structural mechanisms of RTH. In addition to a safe template, our structural and functional study reveals key structural features that define specific recognition of ligands by mutant THRβ and provides structural mechanisms for designing selective and potent ligands of THRβ for the treatment of RTH.

The physiological function of roxadustat has been linked to HIF-PH signaling pathways. Since roxadustat also interacts with THRs, the structural mechanism may provide a basis for designing roxadustat-based compounds that can be used more specifically either for THR- or HIF-PH-regulated diseases, or for a combinatorial therapy. The beneficial and side effects arising from the cross-interaction with each target can be optimized by designing new roxadustat-based compounds with more selectivity. Further elucidation of these two disparate signaling pathways of THR and HIF should reveal specific molecular basis for pharmacological potentials of roxadustat.

Notably, the allosteric regulation has been crucial in regulating receptor function and signal transduction, from inter-molecular domain interactions to intramolecular interactions among DNA (Christopoulos et al., 2014, Putcha and Fernandez, 2009), although the detailed structural mechanisms still need to be further elucidated. Given the highly conserved nature of the ligand-binding pockets, the understanding of the allosterism will reveal more insights into the mechanisms for fine-tuning nuclear receptor function by small molecules, which may lead to a new drug-design strategy targeting allosteric and function-selective sites in modulating nuclear receptor activity (Fernandez, 2018).

### Limitations of the Study

Here we identified a small molecule tool enabling the first probe into the structural mechanisms of RTH and further revealed a molecular mechanism for the defective ligand binding of RTH, thus providing a structural template for designing small molecules in treating RTH. Since the study is based on biochemical and crystallographic assays, the pharmacological potentials of the ligands on *in vivo* animal models will be the subject of future studies.

## Methods

All methods can be found in the accompanying Transparent Methods supplemental file.

## Supplemental Information

Document S1. Transparent Methods, Figures S1–S6, and Table S1
