# Supplementary material for: Revealing a Mutant-Induced Receptor Allosteric Mechanism for the Thyroid Hormone Resistance
Source: iScience. 2019 Oct 2;20:489–96. doi: 10.1016/j.isci.2019.10.002 (PMC6806671; doi:10.1016/j.isci.2019.10.002)
Supplement: Document S1. Transparent Methods, Figures S1–S6, and Table S1 [file mmc1.pdf]

ISCI, Volume *20*

Supplemental Information

Revealing a Mutant-Induced Receptor Allosteric Mechanism for the Thyroid Hormone Resistance

Benqiang Yao, Yijuan Wei, Shuchi Zhang, Siyu Tian, Shuangshuang Xu, Rui Wang, Weili Zheng, and Yong Li

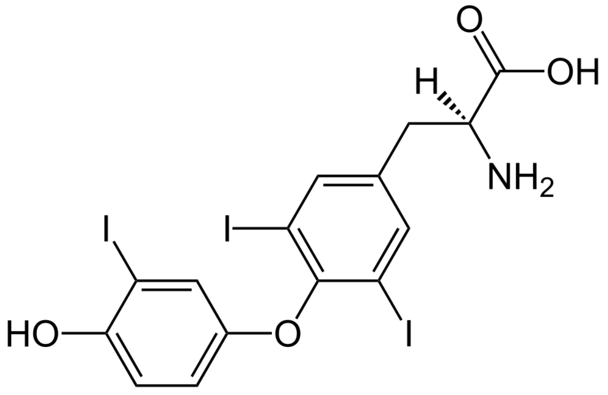

Triiodothyronine (T3)

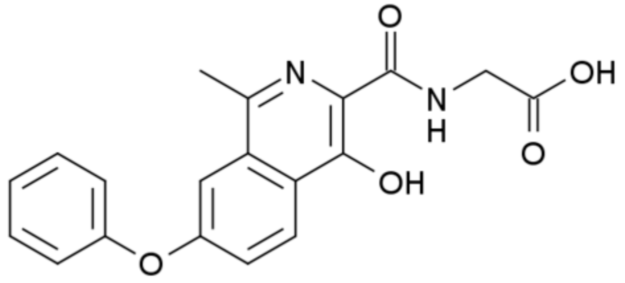

Roxadustat (FG-4592)

Figure. S1. Chemical structures of roxadustat and T3, related to Figure 1. Roxadustat (FG-4592) is a potent hypoxia-inducible factor (HIF) prolyl hydroxylase inhibitor (PHI) for the treatment of anemia, Triiodothyronine (T3) is a physiological ligand for THRs.

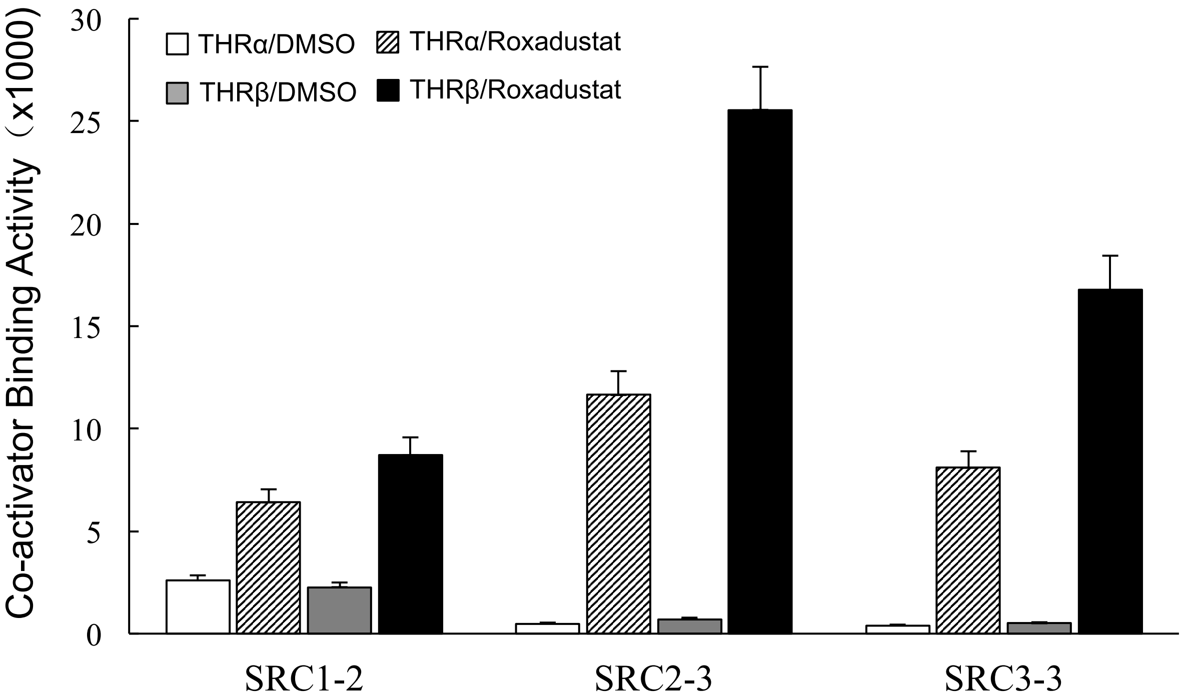

Figure. S2. Roxadustat promotes the interaction of co-activator LXXLL motifs with THRs, related to Figure 1. Modulation of the interaction of THR LBDs with various co-activator LXXLL motifs motifs in response to 1 μM roxadustat was shown by AlphaScreen assays. The peptide sequences are listed in experimental procedures. Values are the means±SD of three independent.

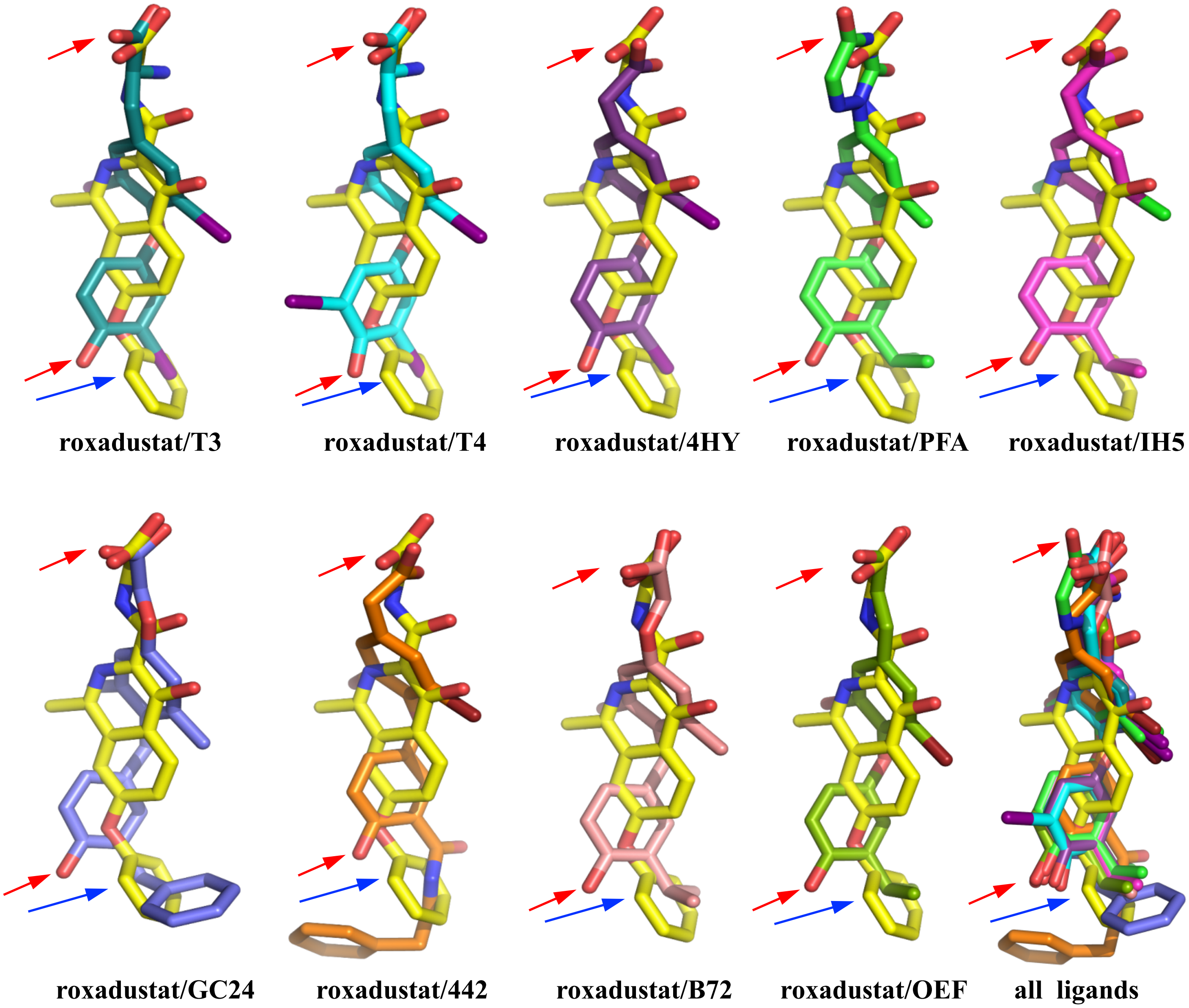

Figure. S3. Superposition of THRβ-bound roxadustat with various THRβ-bound ligands available at PDB, related to Figure 2. Roxadustat (yellow) shares the conserved carboxyl head but not the hydroxy tail groups (both are indicated by red arrows) of THR ligands. The unique hydrophobic benzyl extension of roxadustat is indicated by a blue arrow.

A

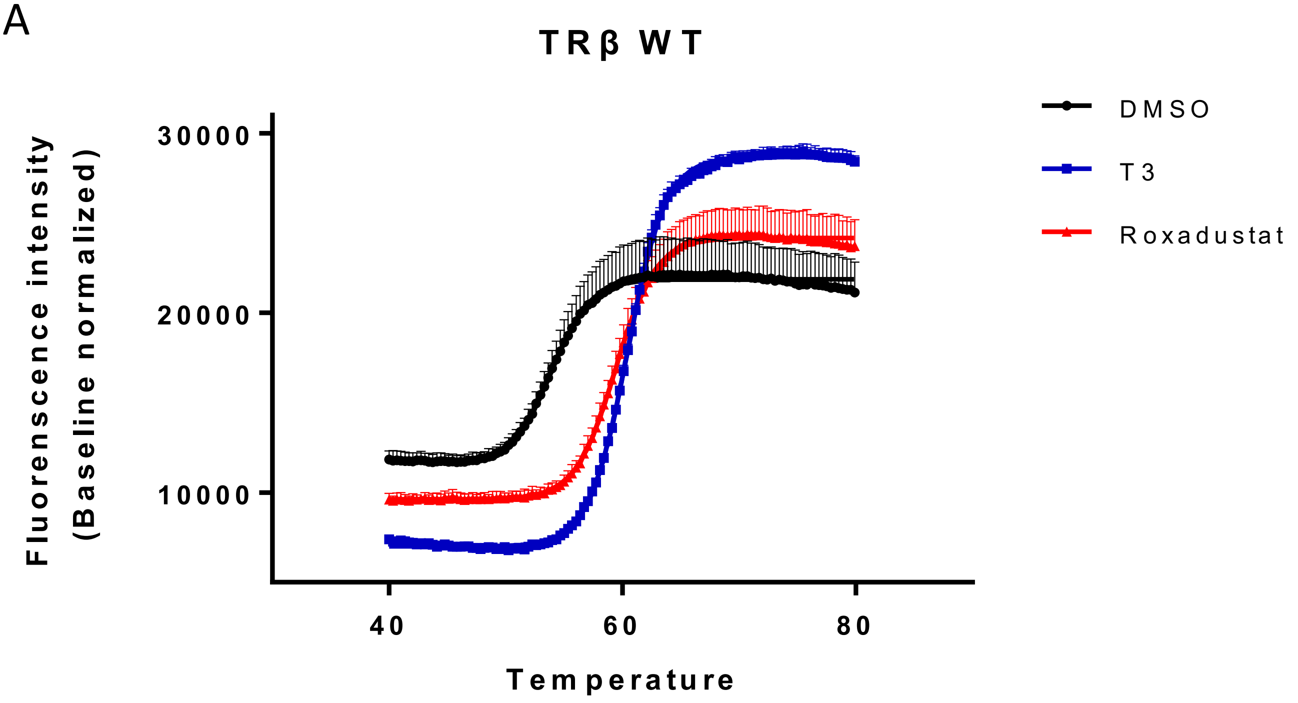

B

Compound	TRβ WT		TRβ V264D		TRβ H435L		TRβ R438W		TRβ R438H	
	Mean Tm(℃)	ΔTm(℃)	Mean Tm(℃)	ΔTm(℃)	Mean Tm(℃)	ΔTm(℃)	Mean Tm(℃)	ΔTm(℃)	Mean Tm(℃)	ΔTm(℃)
DMSO	53.5	0	56	0	63.5	0	54	0	52.5	0
T3	61.5	8	57.5	1.5	64	0.5	56.5	2.5	55	3.5
Roxadustat	59.5	6	61.5	5.5	68	4.5	60	6	58.5	6

Figure S4. Roxadustat significantly improved the thermostability of TRβ, related to Figure 4. (A) Thermal shift assay fluorescence signals obtained for wildtype TRβ/SRC2-3 with and without ligands. Tm values of TRβ/SRC2-3 were shifted 8℃ and 6℃ by T3 and roxadustat, respectively, compared to DMSO. (B) Thermostability characterization of the interactions between TRβ mutants and ligands . Data represent the mean ± SD (n=3). Representative results from three independent experiments are shown.

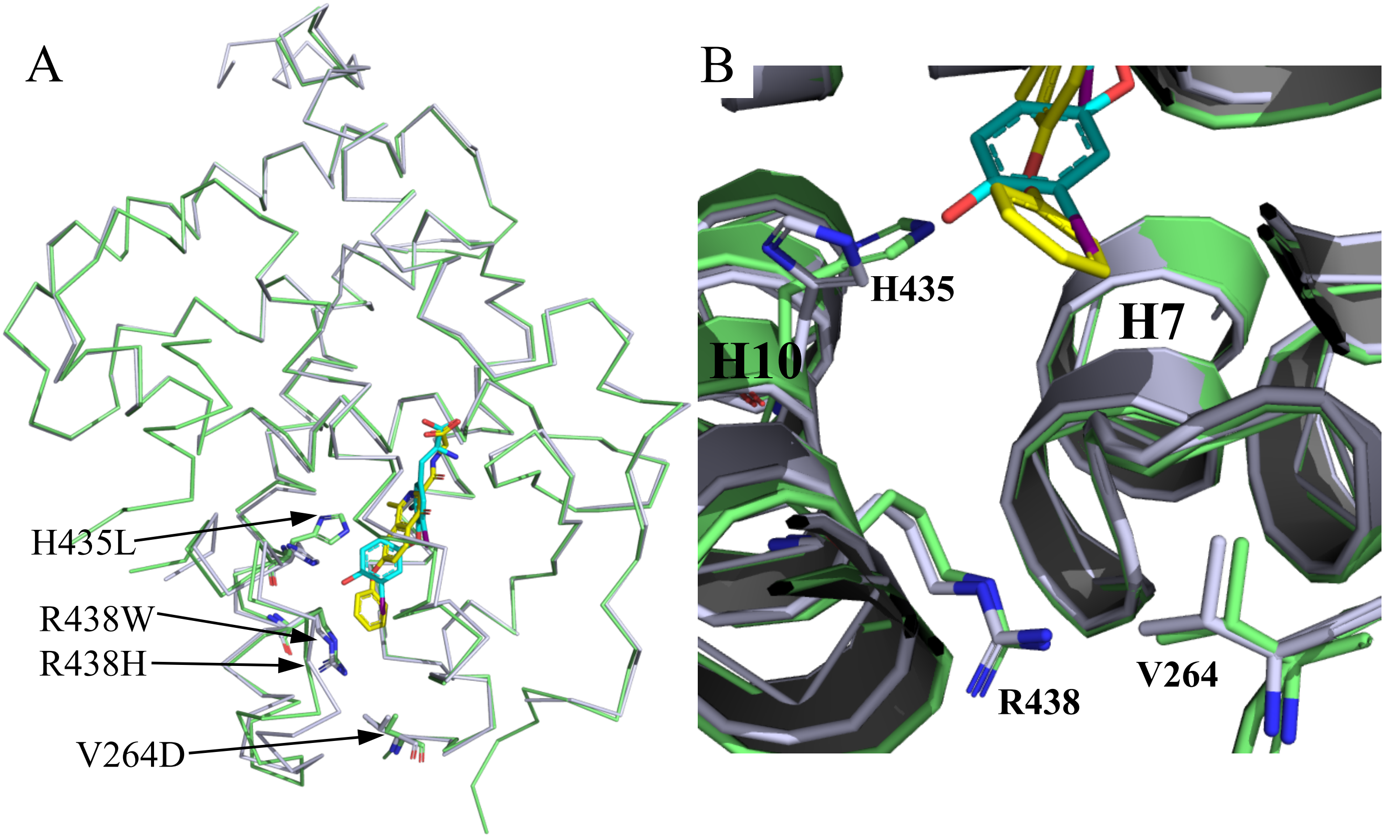

Figure S5. The locations of the 4 THRβ mutations associated with thyroid hormone resistance, related to Figure 4. An overlay of the T3-bound THRβ (light blue) with the roxadustat-bound THRβ (lime) shown in ribbon (A) and cartoon (B) representations, respectively, with T3 and roxadustat shown in cyan and yellow, respectively. The wildtype residues are shown in sticks.

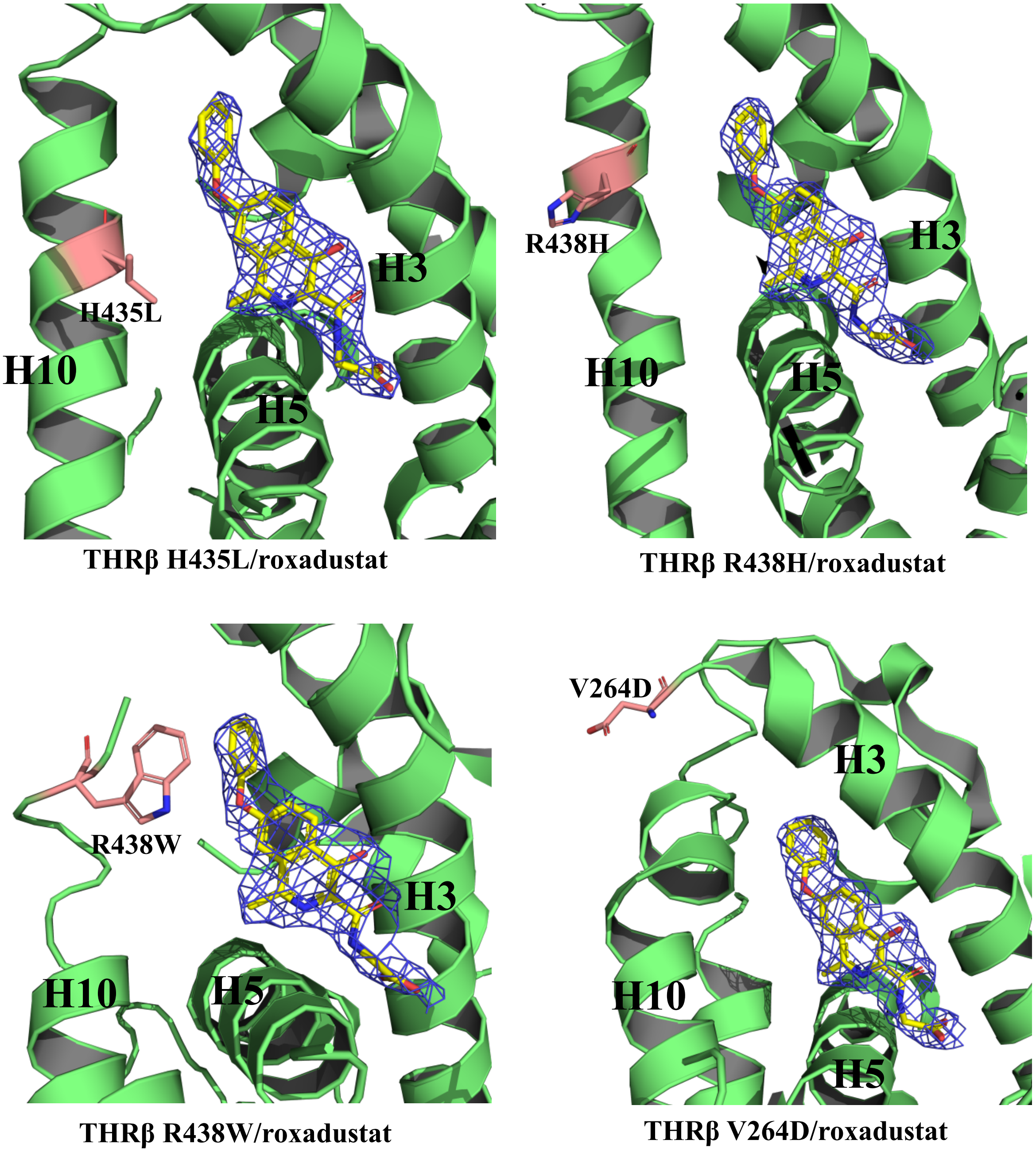

Figure S6. Structural validation of the binding of roxadustat to four THRβ mutants, related to Figure 5. The structure of roxadustat bound with THRβ mutants in cartoon representation. THRβ LBD is colored in lime. The bound roxadustat is shown in stick representation with carbon, nitrogen and oxygen atoms depicted in yellow, blue and red, respectively. 2Fo-Fc electron density map (1.0 σ) showing the bound roxadustat. The mutant residues are shown in salmon red.

Table S1. Data collection and refinement statistics, related to Figures 1 and 5.

Parameters / Complexes	TRβ WT /Roxadustat/SRC2-3	TRβ V264D /Roxadustat/SRC2-3	TRβH435L /Roxadustat/SRC2-3	TRβR438W /Roxadustat/SRC2-3	TRβR438H /Roxadustat/SRC2-3
**PDB ID**	**6KKB**	**6KNU**	**6KKE**	**6KNV**	**6KNW**
**Data collection**					
Space group	P 41 21 2	P 43 21 2	P 43 21 2	P 43 21 2	P 43 21 2
Cell dimensions					
a, b, c (Å)	105.666 105.666 56.950	109.834 109.834 57.524	106.732 106.732 57.079	106.151 106.151 56.624	106.301 106.301 57.353
α, β, γ (°)	90.000 90.000 90.000	90.000 90.000 90.000	90.000 90.000 90.000	90.000 90.000 90.000	90.000 90.000 90.000
Resolution (Å)	50.00-1.72 (1.72-1.69)[a]	49.12-2.40 (2.53-2.40)[a]	70.47-2.57 (2.61-2.57)[a]	50.00-2.80 (2.94-2.80)[a]	75.17-2.67(2.72-2.67)[a]
Rsym [b]	0.156 (0.442)	0.096 (1.458)	0.272 (4.032)	0.250 (1.242)	0.080 (0.550)
I/σ	53.292 (9.800)	15.2(2.9)	18.72(2.39)	4.26 (1.231)	39.07 (10.67)
Completeness (%)	99.01 (97.30)	95.10 (99.90)	99.90 (99.60)	91.60 (95.80)	100.00 (100.00)
Redundancy	25.840 (26.100)	9.9 (11.5)	11.3 (9.3)	3.2 (3.5)	23.7(25.1)
**Refinement**					
Resolution (Å)	50 - 1.70	49.12 - 2.70	29.60- 2.58	50.00-2.80	28.98- 2.67
No. reflections	47231	10124	10192	10613	9818
Rwork / R_{free} [c]	16.31/19.73	17.73/23.12	18.42/21.67	19.39/26.48	17.49/22.94
No. atoms					
Protein	1986	2004	2019	1957	2020
Ligand/ion	26	26	26	26	26
Water	391	56	41	60	38
B factors					
Protein	8.490	61.803	41.864	53.258	41.585
Ligand/ion	13.850	47.828	39.633	52.327	34.905
Water	31.472	49.717	41.210	52.518	36.651
R.m.s.d. [d]					
Bond lengths (Å)	0.0272	0.0087	0.0091	0.0085	0.0080
Bond angles (°)	2.3345	1.0240	1.0730	1.0240	0.9140

[a] Values in parentheses are for highest resolution shell.

[b] $R_{sym}=\sum \mid Iavg - Ii \mid / \sum L$

[c] Rfactor=$\sum |Fp-F_{Pcalc}| / \sum F_{P}$, where F_p and F_{pcalc} are observed and calculated structure factors, R_{free} was calculated from a randomly chosen 8% of reflections excluded from refinement, and R_{factor} was calculated for the remaining 92% of reflections.

[d] R.m.s.d. is the root mean square deviation from ideal geometry.

Transparent Methods

Protein preparation

Mutant human THRβ LBDs were prepared using the QuikChange site-directed mutagenesis kit (Stratagene, La Jolla, CA, USA) with wild-type THRβ LBD (residues 202-461) as the template. The human THRα LBD (residues 148-410), human THRβ LBD and mutant THRβ LBDs were expressed as an amino-terminal 6*His fusion protein from the expression vector pET24a (Novagen, Madison, WI, USA), respectively. BL21 (DE3) cells transformed with expression plasmids were grown in lysogeny broth at 30 ºC to an OD600 of 0.8 and induced with 0.5mM isopropyl isopropyl β-D-thiogalactoside (IPTG) at 22 ºC for 6 h. Cells were collected and sonicated in extraction buffer (20mM Tris pH 8.0, 150mM NaCl, 10% glycerol and 25mM imidazole) on ice. The lysate was centrifuged at 20,000 r.p.m. for 30 min and the supernatant was loaded on a 5-ml NiSO4-loaded HiTrap HP column (GE Healthcare, Piscataway, NJ, USA). The column was washed with extraction buffer and the protein was eluted with a gradient of 25–500mM imidazole. The THR LBDs were further purified with a Q-Sepharose column (GE Healthcare, Piscataway, NJ, USA), followed by gel filtration using a HiLoad 26/600 Superdex 200 column (GE Healthcare, Piscataway, NJ, USA). To prepare the wild type and mutant THRβ LBD protein-ligand complexes, we added a fivefold molar excess of roxadustat (TargetMol, China) and a two-fold molar of a SRC2-3 peptide (ENALLRYLLDKD) to the purified protein, followed by filter concentration to 10 mg mL^{-1}.

Coregulator Binding Assays

The binding of the various coregulator peptide motifs to THR LBDs in response to ligands was determined by AlphaScreen assays using a hexahistidine detection kit from Perkins-Elmer as described before *(1)*. The experiments were conducted with approximately 20-40 nM receptor LBDs and 20 nM biotinylated coregulator peptides in the presence of 5 μg mL^{-1} donor and acceptor beads in a buffer containing 50 mM MOPS, 50 mM NaF, 0.05 mM CHAPS, and 0.1 mg mL-1 bovine serum albumin, all adjusted to a pH of 7.4. The peptides with an N-terminal biotinylation are listed below.

SRC1-2, SPSSHSSLTERHKILHRLLQEGSP;

SRC2-3, QEPVSPKKKENALLRYLLDKDDTKD;

SRC3-3, PDAASKHKQLSELLRGGSG.

Crystallization and structure determination

The crystals of THRβ LBD/roxadustat/SRC2-3 complex were grown at room temperature in hanging drops containing 1.0 μL of the above protein-peptide solutions and 1.0 μL of well buffer containing 0.2 M sodium citrate and 20% polyethylene glycol 3350. The crystals of THRβ(V264D)/roxadustat/SRC2-3 complex were grown in well buffer contained 0.1 M Sodium acetate trihydrate pH 4.6 and 2.0 M Sodium formate. The crystals of THRβ(H435L)/roxadustat/SRC2-3 complex were grown in well buffer contained 0.2 M Sodium tartrate dibasic dihydrate and 20% w/v Polyethylene glycol 3,350. The crystals of THRβ(R438H)/roxadustat/SRC2-3 complex were grown in well buffer contained 100mM NaCl and 20% PEG 4000. The crystals of THRβ(R438W)/roxadustat/SRC2-3 complex were grown in well buffer contained 0.1 M Sodium cacodylate trihydrate pH 6.5 and 1.4 M Sodium acetate trihydrate. These crystals appeared within 1 day and grew to their full size within 2-3 days at room temperature. All crystals were directly flash-frozen in liquid nitrogen for data collection. The observed reflections were reduced, merged and scaled with DENZO and SCALEPACK in the HKL2000 package *(2)*. The

structures were determined by molecular replacement in the CCP4 suite. Manual model building was carried out with Coot *(3)*, followed by Refmac5 refinement in the CCP4 suite.

Transient transfection assay

HEK-293T cells (ATCC) were maintained in Dulbecco's Modified Eagle Medium (DMEM) containing 10% fetal bovine serum and were transiently transfected using Lipofectamine 2000 (Invitrogen). All mutant THR plasmids were created using the Quick-Change Site-Directed Mutagenesis Kit (Stratagene). Before 24 h of transfection, cells were plated in 24-well plates at a density of 5×10^4 cells per well. The cells were transfected with 200 ng Gal4-LBDs of various THRs, respectively, together with 200 ng of pG5Luc reporter (Promega). Ligands were added 5 h after transfection. Cells were harvested 24 h later for luciferase assays with a dual-luciferase reporter assay system (Promega). The luciferase activities were normalized to Renilla activity as an internal control.

Thermal stability analysis.

Thermostability analysis used ABI 7500 Fast, an RT-PCR instrument, as described before *(4-5)*. Briefly, purified protein were preincubated 2 hours with two-fold molar coregulator peptides and DMSO or five-fold molar excess T3 or FG-4592, respectively. Standard assay conditions (20 µL) contain 12.5 µL protein complex (24uM), 7.5ul Protein Thermal Shift Tm buffer and 1/1000 volume of ROX dye (Protein Thermal ShiftTM Dye Kit, Applied Biosystems, USA). Thermal shift assays were performed in 96-well PCR plates which were heated from 40°C to 80°C at a rate of 1°C/min and the fluorescence data were obtained on 7500 fast real-time PCR instrument. The 50% of maximum temperature (Tm) value of protein/ cofactor/ compound was analyzed by GraphPad Prism 7 and fitted using Boltzmann sigmoid curves. All experiments were performed in triplicates.

Supplemental References

1. Jin L*, et al.* (2013) The antiparasitic drug ivermectin is a novel FXR ligand that regulates metabolism. *Nat Commun* 4:1937.
2. Otwinowski Z & Minor W (1997) Processing of X-ray diffraction data collected in oscillation mode. *Methods Enzymol* 276:307-326.
3. Emsley P & Cowtan K (2004) Coot: model-building tools for molecular graphics. *Acta Crystallogr D Biol Crystallogr* 60(Pt 12 Pt 1):2126-2132.
4. Padyana, A. K. et al. (2019) Structure and inhibition mechanism of the catalytic domain of human squalene epoxidase. Nature communications 10, 97.
5. Iwata, H. et al. (2012) A back-to-front fragment-based drug design search strategy targeting the DFG-out pocket of protein tyrosine kinases. ACS medicinal chemistry letters 3, 342-346.
